# Statin use and risk of Parkinson’s disease among older adults in Japan: a nested case–control study using the Longevity Improvement and Fair Evidence study

**DOI:** 10.1093/braincomms/fcae195

**Published:** 2024-06-04

**Authors:** Sanyu Ge, Ling Zha, Yasuyoshi Kimura, Yoshimitsu Shimomura, Masayo Komatsu, Yasufumi Gon, Sho Komukai, Fumiko Murata, Megumi Maeda, Kosuke Kiyohara, Tomotaka Sobue, Tetsuhisa Kitamura, Haruhisa Fukuda

**Affiliations:** Division of Environmental Medicine and Population Sciences, Department of Social Medicine, Graduate School of Medicine, Osaka University, Suita, 565-0871, Osaka, Japan; Division of Environmental Medicine and Population Sciences, Department of Social Medicine, Graduate School of Medicine, Osaka University, Suita, 565-0871, Osaka, Japan; Department of Neurology, Graduate School of Medicine, Osaka University, Suita, Osaka, 565-0871, Japan; Division of Environmental Medicine and Population Sciences, Department of Social Medicine, Graduate School of Medicine, Osaka University, Suita, 565-0871, Osaka, Japan; Division of Environmental Medicine and Population Sciences, Department of Social Medicine, Graduate School of Medicine, Osaka University, Suita, 565-0871, Osaka, Japan; Department of Neurology, Graduate School of Medicine, Osaka University, Suita, Osaka, 565-0871, Japan; Biomedical Statistics, Department of Integrated Medicine, Graduate School of Medicine, Osaka University, Suita, Osaka, 565-0871, Japan; Department of Health Care Administration and Management, Kyushu University Graduate School of Medical Sciences, Fukuoka, 812-0054, Japan; Department of Health Care Administration and Management, Kyushu University Graduate School of Medical Sciences, Fukuoka, 812-0054, Japan; Department of Food Science, Faculty of Home Economics, Otsuma Women’s University, Tokyo, 102-8357, Japan; Division of Environmental Medicine and Population Sciences, Department of Social Medicine, Graduate School of Medicine, Osaka University, Suita, 565-0871, Osaka, Japan; Division of Environmental Medicine and Population Sciences, Department of Social Medicine, Graduate School of Medicine, Osaka University, Suita, 565-0871, Osaka, Japan; Department of Health Care Administration and Management, Kyushu University Graduate School of Medical Sciences, Fukuoka, 812-0054, Japan

**Keywords:** statin, Parkinson’s disease, longevity improvement and fair evidence study, Japanese older adults

## Abstract

The association between statin use and the risk of Parkinson’s disease remains inconclusive, particularly in Japan’s super-ageing society. This study aimed to investigate the potential association between statin use and the risk of Parkinson’s disease among Japanese participants aged ≥65 years. We used data from the Longevity Improvement and Fair Evidence Study, which included medical and long-term care claim data from April 2014 to December 2020 across 17 municipalities. Using a nested case–control design, we matched one case to five controls based on age, sex, municipality and cohort entry year. A conditional logistic regression model was used to estimate the odds ratios with 95% confidence intervals. Among the 56 186 participants (9397 cases and 46 789 controls), 53.6% were women. The inverse association between statin use and Parkinson’s disease risk was significant after adjusting for multiple variables (odds ratio: 0.61; 95% confidence interval: 0.56–0.66). Compared with non-users, the dose analysis revealed varying odds ratios: 1.30 (1.12–1.52) for 1–30 total standard daily doses, 0.77 (0.64–0.92) for 31–90 total standard daily doses, 0.62 (0.52–0.75) for 91–180 total standard daily doses and 0.30 (0.25–0.35) for >180 total standard daily doses. Statin use among older Japanese adults was associated with a decreased risk of Parkinson’s disease. Notably, lower cumulative statin doses were associated with an elevated risk of Parkinson’s disease, whereas higher cumulative doses exhibited protective effects against Parkinson’s disease development.

## Teaser

Ge *et al*. report that statin use was associated with a decreased risk of Parkinson’s disease among older Japanese adults, based on claim data. Lower cumulative doses of statins were associated with a higher risk of Parkinson’s disease, while higher cumulative doses had a protective effect against Parkinson’s disease.

## Graphical Abstract

**Graphical Abstract:**
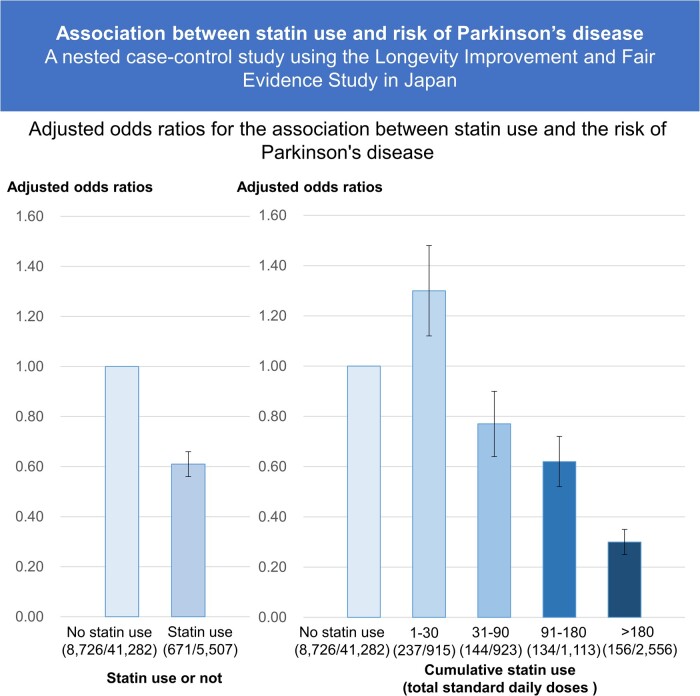

## Introduction

Parkinson’s disease is the second most prevalent neurodegenerative disease associated with illness.^1^ Although the incidence is relatively low until the age of 50 years,^2^ it exhibits an upward trend with advancing age.^3^ The onset of Parkinson’s disease has been associated with genetic, environmental and behavioural factors, as well as certain medications, such as statins and non-steroidal anti-inflammatory drugs.^2^

Statins are primarily prescribed to reduce blood cholesterol levels.^4^  *In vitro* and *in vivo* studies using Parkinson’s disease models suggested a possible protective effect of statins against Parkinson’s disease.^5-9^ However, recent observational studies have yielded conflicting results regarding the relationship between statins and Parkinson’s disease.^10-18^ Studies conducted in the USA and Korea have suggested an increased risk of Parkinson’s disease with statin use,^13-15,17^ while studies performed in Taiwan and France have suggested a protective effect.^10,16,18^ Moreover, a relationship between cumulative statin dosage and the risk of Parkinson’s disease has been observed in French, Taiwanese and Korean studies. However, these associations demonstrated a significant inconsistency.^14-16,18^ For instance, the French study suggests an association between an average daily lipophilic statin dose of ≥0.67 defined daily doses (DDDs) and a reduction in Parkinson’s disease. A Taiwanese study suggested that a cumulative statin intake of >250 days was associated with a decreased risk of Parkinson’s disease. Conversely, a Korean study demonstrated an increased risk of Parkinson’s disease with doses lasting <365 days, whereas days exceeding this threshold did not show a similar association. In addition, the association between the types of statins and the risk of Parkinson’s disease still remains inconclusive. Some studies have shown that lipophilic statins, which can more easily cross the blood–brain barrier than hydrophilic statins,^19^ may have a protective effect against Parkinson’s disease.^18,20^ However, other studies have not found any difference between lipophilic and hydrophilic statins regarding their association with the risk of Parkinson’s disease.^14-16^ Thus, the association between statin use, including the cumulative dose and types of statins, and Parkinson’s disease remains inconclusive and has not been sufficiently investigated in Japan, a country with one of the most aged populations.

Herein, the present study aimed to investigate the association between statin use and Parkinson’s disease using large-scale claim data from the Longevity Improvement and Fair Evidence (LIFE) study. Unlike previous studies,^12,13,17^ we specifically investigated the different types of statins and the effect of long-term statin use on Parkinson’s disease.

## Materials and methods

### Study data

This nested case–control study used data from the LIFE study. The detailed design of the LIFE study and the demographic characteristics of the participants have been documented in the literature. Briefly, municipalities participating in the LIFE study contributed data from government-administered health insurance enrolees and recipients of public assistance, including individuals across all age groups and various disease types.^21^ This study linked medical and long-term care (LTC) claim data, health check-up data, vaccination records, residence-related information and income-related information. Our study utilized data from 17 different municipalities, encompassing 1 694 083 individuals, from April 2014 to December 2020 and included both medical and LTC information.

The study was approved by the Kyushu University Institutional Review Board for Clinical Research (approval no. 22114) and the Osaka University Institutional Review Board (approval no. 21107).

### Study participants

The number of participants in each municipality, the number of patients in each municipality and the data period for analysis are listed in Supplementary Table 1. Our study used data from April 2014, with the exact start dates varying across municipalities according to their participation. To establish the baseline covariables, the 6-month period from the initial claims record (covering any medical service or treatment) was set as the lookback period.^22^ Therefore, the cohort entry time for our study was defined as the first day after the lookback period (specifically, the first day of the seventh month after the initial claims record). The conclusion of the cohort evaluation was determined as the earliest among the following time points: the time of the last claims record, the time of Parkinson’s disease diagnosis and December 2020. A detailed graphical depiction of the study design is provided in Supplementary Fig. 1.

**Figure 1:**
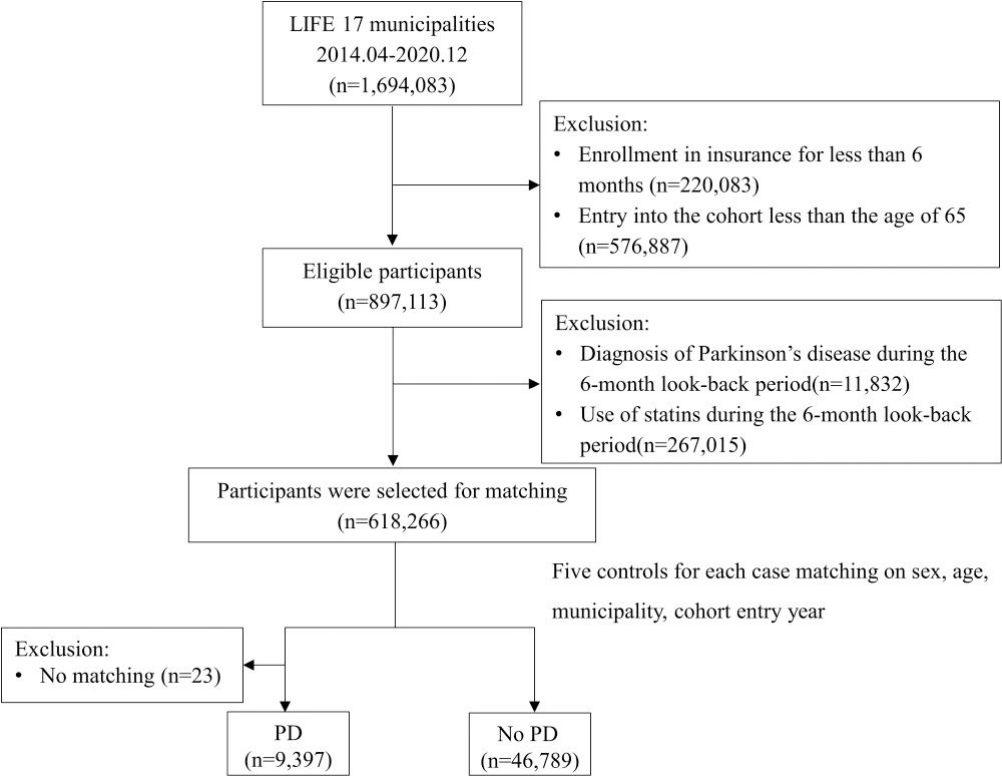
**Flow chart of the selection of study participants and cases and their controls.** For each case, five event-free controls were randomly selected based on the identification date. The matching criteria were age, date of cohort entry, sex, municipality (17 municipalities) and year of cohort entry (2014, 2015, 2016, 2017, 2018, 2019 or 2020). PD, Parkinson’s disease; LIFE, Longevity Improvement and Fair Evidence.

A flow chart of the study participants is shown in Fig. 1. Participants who had been enrolled in insurance for <6 months (*n* = 220 083), aged <65 years on the cohort entry date (*n* = 576 887), diagnosed with Parkinson’s disease (*n* = 11 832), or prescribed with statins (*n* = 267 015) during the lookback period were excluded. Finally, only 618 266 participants were selected for matching.

### Identification of cases and controls

A nested case–control study was conducted using data from the LIFE study carried out between 2014 and 2020. Parkinson’s disease was defined using the 10th edition of the International Classification of Diseases and Related Health Problems (ICD-10: G20). A 1:5 case–control matching was performed. Controls were matched to cases based on age at cohort entry, sex, municipality (17 municipalities) and year of cohort entry (2014, 2015, 2016, 2017, 2018, 2019 or 2020). The index date for the cases was defined as the date of diagnosis, while controls were assigned an index date corresponding to the date of diagnosis of their matched cases. In the final analysis, 9397 cases and 46 789 controls were included after excluding cases with no corresponding matches (*n* = 23).

### Exposure assessment

Data regarding the prescribed statins were obtained from the medical claims record. In our study, statins primarily comprised six distinct medications (atorvastatin, fluvastatin, pitavastatin, pravastatin, rosuvastatin and simvastatin) along with certain statin combinations (comprehensive details are available in Supplementary Table 3). The primary exposure variable in this study was statin use. In addition, we computed the cumulative daily statin dosage for each participant from the time of cohort entry to the index date. The specific calculation process involved the following steps:

1. We calculated the total dosage of each prescribed statin and determined the corresponding quantity of statin components for combination drugs.
2. We standardized the total dosage of each prescribed statin using the DDD provided on the World Health Organization website.^23^
3. Subsequently, we summed all the standardized values for the same patient to obtain the total standardized daily dose (TSDD).

This definition of cumulative daily statin dosage aligned with the methodology used in several previous studies.^14,24,25^ Cumulative exposure was categorized into five groups: no use (Category 1), 1–30 TSDDs (Category 2), 31–90 TSDDs (Category 3), 90–180 TSDDs (Category 4) and >180 TSDDs (Category 5). Statins were further classified into lipophilic and hydrophilic. Pravastatin and rosuvastatin were classified as hydrophilic statins, whereas atorvastatin, fluvastatin, pitavastatin and simvastatin were categorized as lipophilic statins.

### Covariates

The individual baseline covariates were established during the lookback period. Comorbidities were determined using the Elixhauser Comorbidity Index based on ICD-10 (comprehensive details are available in Supplementary Table 5).^21,26,27^ According to a previous study,^25^ congestive heart failure (CHF), cardiac arrhythmias, valvular disease, pulmonary circulation disorders, peripheral vascular disease, hypertension, chronic pulmonary disease, diabetes, renal failure, liver disease, cancer, psychosis and depression were selected as covariates. Data on LTC were obtained from the LTC insurance system. The municipality-certified levels of LTC needs were determined by assessing the presence or absence of disabilities and instrumental activities of daily living. Based on the findings of previous studies on the LIFE study, LTC needs were categorized into no need, support need Level 1–2 and care need Level 1–5.^25,28^ The need for LTC was defined as the highest level of care during the lookback period. Furthermore, the number of outpatient visits during the lookback period was calculated and used as a covariate to assess an individual’s behaviour during the healthcare provider visit.

### Statistical analysis

The characteristics of the control and case groups were compared. Conditional logistic regression was used to analyse the association between statin use and the risk of developing Parkinson’s disease. Model 1 was not adjusted. Model 2 was adjusted for comorbidities, such as CHF (yes or no), cardiac arrhythmias (yes or no), valvular disease (yes or no), pulmonary circulation disorders (yes or no), peripheral vascular disease (yes or no), hypertension (yes or no), chronic pulmonary disease (yes or no), diabetes (yes or no), renal failure(yes or no), liver disease (yes or no), cancer (yes or no), psychosis (yes or no) and depression (yes or no); the number of months with outpatient visits over a 6-month period (0, 1, 2, 3, 4, 5 and 6); and the LTC need levels (no support, Support 1–2, Care 1, Care 2, Care 3, Care 4 and Care 5). Two stratified analyses were performed by sex (male and female) and age group (65–74, 75–84 and >85 years). To investigate the interaction between statin use and sex or age groups, the product terms for statin use and sex or age categories were introduced into Model 2. The same model was established to assess the association between cumulative statin use and Parkinson’s disease risk. Considering the varying entry timing, we also conducted a stratified analysis based on the time of enrolment. In this study, we categorized eligible patients into two cohorts according to their period of entry: one from 2014 to 2016 and the other from 2017 to 2020.

The association between the different types of statins and Parkinson’s disease was also examined. Patients using other types of statins were excluded from the analysis of the association between specific statins and Parkinson’s disease risk. This exclusion was implemented to ensure that the statin-free group consisted of patients who had never used any type of statin. Following a 1:5 re-matching of the cases and controls, we repeated our previous analyses.

Three sensitivity analyses were conducted. First, individuals using more than one statin were excluded to reduce the potential effects of concomitant use of multiple statins. Second, individuals with a follow-up period of <180 or 365 days were also excluded. Third, Parkinson’s disease was defined using the ICD-10 and the medical records. Patients who were diagnosed with G20 disease and received medications for Parkinson’s disease in the same or subsequent months were included in this analysis. The specific medications used to treat Parkinson’s disease are listed in Supplementary Table 4.

Furthermore, the *E*-values were calculated to evaluate the robustness of the results against potential residual or unmeasured confounders. The *E*-value represents the minimum strength of association that an unmeasured confounder would need to have with both exposure and outcome variables.^29^

All reported *P*-values were two-sided, and a *P-*value of <0.05 was considered significant. *E*-values were calculated using an *E*-value calculator available on a specific website.^29-31^ All statistical analyses were conducted using the Stata software (version 16.0; Stat Corporation, College Station, TX, USA).

## Results

A total of 9397 cases and 46 789 controls were analysed, of whom 53.6% were women (5035 cases and 25 072 controls). No differences were observed in the matching factors (including sex, age at cohort entry, municipality or year of cohort entry) between the case and control groups (Supplementary Table 2). Table 1 shows that the case group exhibited a higher prevalence of CHF, cardiac arrhythmias, peripheral vascular disease, renal failure, psychosis and depression compared with the control group. The control group had a lower level of LTC and a higher frequency of monthly outpatient visits compared with the case group.

**Table 1:** Characteristics of Parkinson’s disease cases and their controls

	Participants, *n* (%)		Standardized difference
Characteristic	Controls	Case (PD)	
*N*	46 789	9397	
*Comorbidities at lookback period (yes), n (%)*			
Congestive heart failure	7566 (16.2)	1746 (18.6)	0.06
Cardiac arrhythmias	7666 (16.4)	1738 (18.5)	0.06
Valvular disease	3010 (6.4)	575 (6.1)	0.01
Pulmonary circulation disorders	192 (0.4)	37 (0.4)	<0.01
Peripheral vascular disorders	5280 (11.3)	1166 (12.4)	0.03
Hypertension	26 882 (57.5)	5341 (56.8)	0.01
Chronic pulmonary disease	9317 (19.9)	1935 (20.6)	0.02
Diabetes	3110 (6.6)	638 (6.8)	0.01
Renal failure	2132 (4.6)	518 (5.5)	0.04
Liver disease	9075 (19.4)	1896 (20.2)	0.02
Cancer	6286 (13.4)	1224 (13.0)	0.01
Psychosis	1335 (2.9)	1145 (12.2)	0.36
Depression	2929 (6.3)	1489 (15.8)	0.31
*LTC needs, n (%)*			0.34
No	38 248 (81.7)	6345 (67.5)	
Support 1–2	2571 (5.5)	708 (7.5)	
Care 1	1767 (3.8)	676 (7.2)	
Care 2	1514 (3.2)	618 (6.6)	
Care 3	1101 (2.4)	435 (4.6)	
Care 4	935 (2.0)	371 (3.9)	
Care 5	653 (1.4)	244 (2.6)	
*Number of months with outpatient visit, n (%)*			0.19
0	609 (1.3)	289 (3.1)	
1	195 (0.4)	87 (0.9)	
2	314 (0.7)	106 (1.1)	
3	648 (1.4)	224 (2.4)	
4	1545 (3.3)	397 (4.2)	
5	2843 (6.1)	719 (7.7)	
6	40 635 (86.8)	7575 (80.6)	

PD, Parkinson's disease.

Table 2 presents the association between statin use and the risk of Parkinson’s disease. Model 2 showed that statin use was associated with a decreased risk of Parkinson’s disease [odds ratio (OR): 0.61, 95% confidence interval (CI): 0.56–0.66] compared with non-use. The *E*-value for the observed association estimates between statin use and Parkinson’s disease was 2.66, with the limit of the 95% CI being closest to null at 2.4. Similar outcomes were observed in both sexes [men: 0.62 (0.54–0.70); women: 0.60 (0.54–0.68); *P* for interaction = 0.71] and different age groups [65–74 years: 0.57 (0.49–0.66), 75–84 years: 0.60 (0.53–0.68) and >85 years: 0.73 (0.59–0.92); *P* for interaction = 0.17]. Table 3 demonstrates the association between cumulative statin use and Parkinson’s disease risk. In comparison with non-statin users, the 1–30 TSDD group exhibited an OR of 1.30 (1.12–1.52). In contrast, the 31–90 TSDD group had an OR of 0.77 (0.64–0.92), the 91–180 TSDD group had an OR of 0.62 (0.52–0.75) and >180 TSDD group had an OR of 0.30 (0.25–0.35). Overall, higher cumulative statin use was associated with a lower risk of Parkinson’s disease.

**Table 2:** Association between statin use and risk of Parkinson’s disease

			No statin use^a^	Statin use^a^	*P* for interaction
All		Case/control	8726/41 282	671/5507	
		Model 1	Ref.	0.57 (0.53–0.62)	
		Model 2	Ref.	0.61 (0.56–0.66)	
Sex					0.71
	Men	Case/control	4073/19 381	289/2336	
		Model 1	Ref.	0.59 (0.52–0.67)	
		Model 2	Ref.	0.62 (0.54–0.70)	
	Women	Case/control	4653/21 901	382/3171	
		Model 1	Ref.	0.56 (0.50–0.63)	
		Model 2	Ref.	0.60 (0.54–0.68)	
Age					0.17
	65–74	Case/control	2425/11 278	231/1964	
		Model 1	Ref.	0.55 (0.47–0.63)	
		Model 2	Ref.	0.57 (0.49–0.66)	
	74–84	Case/control	4298/20 270	345/2873	
		Model 1	Ref.	0.56 (0.50–0.63)	
		Model 2	Ref.	0.60 (0.53–0.68)	
	85-	Case/control	2003/9734	95/670	
		Model 1	Ref.	0.69 (0.55–0.86)	
		Model 2	Ref.	0.73 (0.59–0.92)	

Model 1: no adjustment. Model 2: adjusted for CHF (yes or no), cardiac arrhythmias (yes or no), valvular disease (yes or no), pulmonary circulation disorders (yes or no), peripheral vascular disorders (yes or no), hypertension (yes or no), chronic pulmonary disease (yes or no), diabetes (yes or no), renal failure (yes or no), liver disease (yes or no), cancer (yes or no), psychoses (yes or no), depression (yes or no), number of months with outpatient visits and LTC needs. ^a^The percentage of statin users was 11.0%, while the percentage of non-users was 89.0%.

**Table 3:** Association between total cumulative use of statin drugs and risk of Parkinson’s disease

	TSDDs of statin	Case/control	Model 1	Model 2
Category 1^a^	0	8726/41 282	Ref.	Ref.
Category 2^a^	1–30	237/915	1.22 (1.05–1.41)	1.30 (1.12–1.52)
Category 3^a^	31–90	144/923	0.73 (0.61–0.87)	0.77 (0.64–0.92)
Category 4^a^	91–180	134/1113	0.57 (0.47–0.68)	0.62 (0.52–0.75)
Category 5^a^	>180	156/2556	0.29 (0.24–0.34)	0.30 (0.25–0.35)

Model 1: no adjustment. Model 2: adjusted for CHF (yes or no), cardiac arrhythmias (yes or no), valvular disease (yes or no), pulmonary circulation disorders (yes or no), peripheral vascular disorders (yes or no), hypertension (yes or no), chronic pulmonary disease (yes or no), diabetes (yes or no), renal failure (yes or no), liver disease (yes or no), cancer (yes or no), psychoses (yes or no), depression (yes or no), number of months with outpatient visits and LTC needs. ^a^The percentages of individuals in each group were as follows: 89.0% (Category 1); 2.0% (Category 2); 2.0% (Category 3); 2.2% (Category 4) and 4.8% (Category 5).

Table 4 shows the association between the risk of Parkinson’s disease and the use of different types of statins. Owing to the limited data on fluvastatin and simvastatin users, no association was found between the use of different types of statins and Parkinson’s disease. Nevertheless, the use of other statins has been associated with a reduced risk of Parkinson’s disease. Supplementary Table 6 analyses confirmed this trend for both lipophilic and hydrophilic statins, both of which showed a reduced risk of Parkinson’s disease.

**Table 4:** Association between specific types of statin use and risk of Parkinson’s disease^a^

	No statin use^b^	Statin use^b^
Atorvastatin		
Case/control	8726/42 979	155/1222
Model 2	Ref.	0.67 (0.56–0.79)
Fluvastatin		
Case/control	8725/43 439	8/27
Model 2	Ref.	1.44 (0.63–3.30)
Pitavastatin		
Case/control	8725/42 955	81/887
Model 2	Ref.	0.49 (0.39–0.62)
Pravastatin		
Case/control	8725/43 254	102/682
Model 2	Ref.	0.79 (0.64–0.98)
Rosuvastatin		
Case/control	8726/42 465	206/2002
Model 2	Ref.	0.54 (0.47–0.63)
Simvastatin		
Case/control	8725/43 428	14/73
Model 2	Ref.	0.99 (0.55–1.79)

Model 2: adjusted for CHF (yes or no), cardiac arrhythmias (yes or no), valvular disease (yes or no), pulmonary circulation disorders (yes or no), peripheral vascular disorders (yes or no), hypertension (yes or no), chronic pulmonary disease (yes or no), diabetes (yes or no), renal failure (yes or no), liver disease (yes or no), cancer (yes or no), psychoses (yes or no), depression (yes or no), number of months with outpatient visits and LTC needs. ^a^When exploring the relationship between atorvastatin and the risk of Parkinson’s disease, we excluded patients who had used other statins. Following this, we conducted an analysis on the cases and controls after applying a 1:5 re-matching procedure. This process was repeated for other statins. ^b^For atorvastatin, the percentage of users was 2.6%, while the percentage of non-users was 97.4%. For fluvastatin, the percentage of users was 0.1%, while the percentage of non-users was 99.9%. For pitavastatin, the percentage of users was 1.8%, while the percentage of non-users was 98.2%. For pravastatin, the percentage of users was 1.5%, while the percentage of non-users was 98.5%. For rosuvastatin, the percentage of users was 4.1%, while the percentage of non-users was 95.9%. For simvastatin, the percentage of users was 0.2%, while the percentage of non-users was 99.8%.

In the sensitivity analysis, which excluded individuals taking multiple statins or individuals with a follow-up period of >180 or 365 days, the results remained unchanged (Supplementary Tables 7–10). In an additional sensitivity analysis, both ICD-10 coding and medications for Parkinson’s disease were employed to define Parkinson’s disease (Supplementary Table 4), leading to the identification of 5942 patients with Parkinson’s disease and 29 607 controls. These fundamental characteristics mirrored those of previous analyses (Supplementary Table 11). Statin use was associated with a decreased occurrence of Parkinson’s disease (Supplementary Table 12). Nevertheless, when examining cumulative statin dosage (Supplementary Table 13), similar to the results of the analysis based on the ICD-10 definition of Parkinson’s disease, the risk of Parkinson’s disease was higher in the 1–30 TSDD group, although this was not significant. Additionally, the incidence of Parkinson’s disease declined with increased cumulative statin use.

There were no differences in the association between statin use, cumulative dose and the risk of Parkinson’s disease between the groups enrolled during 2014–16 and 2017–20, as detailed in Supplementary Tables 14 and 15.

## Discussion

To our knowledge, this study is the first to examine the association between statin use and Parkinson’s disease risk using a population-based administrative claim database in Japan. Our study revealed an association between statin use and a lower risk of Parkinson’s disease in elderly Japanese participants. Furthermore, the analysis of cumulative statin use explored the correlation between cumulative dose and Parkinson’s disease, demonstrating that low cumulative statin use increased the risk of Parkinson’s disease, whereas high cumulative statin usage corresponded to a reduction in Parkinson’s disease risk. This association remained consistent in various statin types. Similar results were obtained in the multiple sensitivity analyses.

In our study, the individuals taking statins had a reduced risk of developing Parkinson’s disease. Notably, this effect remained consistent across sexes (men and women) and age groups (65–74, 75–84 and ≥85 years). Overall, our findings aligned with those of previous studies conducted in France,^18^ Taiwan,^10,16^ the USA (Health Professionals Follow-up and the Nurses’ Health Study)^11^ and Denmark.^32^ Conversely, studies from Korea^14,15^ and the USA (US claim database: MarketScan; Atherosclerosis Risk in Communities Study)^13,17^ indicated a potential adverse association between statin use and Parkinson’s disease. The methodologies differed between our study and two previous case–control studies conducted in the USA.^13,17^ A previous study highlighted the substantial influence of study design on outcomes.^18^ Although the two Korean studies adopted a design similar to that of the present study, their primary observation indicated an increased risk in the short-term statin use group, but no association in long-term use group.^14,15^ In addition, three meta-analyses showed that statin use was associated with a reduced risk of Parkinson’s disease.^33-35^ Our findings further reinforce the inverse association between statin use and the risk of Parkinson’s disease.

Our study revealed an association between a high cumulative dose of statins and a reduced risk of Parkinson’s disease. However, the low cumulative statin dose group demonstrated an increased risk of Parkinson’s disease compared with non-users. This 1–30 TSDD group may comprise subjects diagnosed with hypercholesterolaemia with high low-density lipoprotein cholesterol (LDL-C) and started on statins, which were quickly discontinued possibly due to poor adherence or adverse events. This group may also include patients who were diagnosed with Parkinson’s disease soon after statin therapy for hypercholesterolaemia. The association between serum cholesterol levels and Parkinson’s disease is still controversial. A previous study in Finland suggested that higher blood cholesterol levels were associated with an increased risk of Parkinson’s disease.^36^ However, recent two meta-analyses suggested an inverse association between serum LDL-C levels and Parkinson’s disease risk, although the conclusion regarding the association between Parkinson’s disease risk and serum levels of total cholesterol or triglycerides was inconsistent.^37,38^ Of note, Parkinson’s disease could dysregulate lipid metabolism and vice versa, and prodromal symptoms of Parkinson’s disease may affect serum cholesterol levels. Thus, future studies will be required to elucidate the association among hypercholesterolemia, statin usage and Parkinson’s disease. One possible reason for an increased risk of Parkinson’s disease in this low TSDD group is that this group may comprise patients at the prodromal phase of Parkinson’s disease. On the other hand, a substantial number of Parkinson’s disease patients suffer non-motor symptoms including depression prior to their diagnosis of Parkinson’s disease. This could result in discontinuation of newly prescribed drugs due to poor adherence, leading to an increased number of Parkinson’s disease cases in the low TSDD group. However, we adjusted variables including depression and psychosis in multivariable models to control for the effects of these factors in this study. Conversely, in the higher cumulative dose group, the population was continuously treated with statins and might have better control of hypercholesterolaemia. In addition, statins may exert protective effects such as anti-inflammatory effects against Parkinson’s disease.^39,40^ Consequently, we observed a reduced risk of Parkinson’s disease in this group. This observation reinforces the notion that continuous statin use is associated with a decreased risk of developing Parkinson’s disease.

Several mechanisms can explain the protective effects of statins. Statins can reduce the risk of Parkinson’s disease by lowering cerebral atherosclerosis.^41^ Statin use potentially enhances the survival of dopaminergic neurones, and changes in dopamine levels have been linked to Parkinson’s disease development.^6,42^ Previous animal experiments have demonstrated that statin exhibits anti-inflammatory effects and safeguards dopaminergic nerves by modulating cyclooxygenase-2 and *N*-methyl-D-aspartate.^9,42^ Furthermore, statins suppress pro-inflammatory molecules and microglial activation.^42^ Additionally, they attenuated α-synuclein aggregation, thereby potentially mitigating Parkinson’s disease progression.^5,6^

Although a recent RCT in the UK suggested that simvastatin was ineffective as a disease-modifying therapy in patients with moderately severe Parkinson’s disease,^43^ no RCT reported the use of statins for preventing the development of Parkinson’s disease among older people. Therefore, our study provides evidence that supports the use of statins to prevent Parkinson’s disease. Furthermore, the different cumulative doses of statins might have varying associations with Parkinson’s disease, with high cumulative doses of statins showing a protective effect in preventing Parkinson’s disease. This finding is consistent with those of previous studies, which suggested that discontinuing statin use is associated with an increased risk of Parkinson’s disease.^20^ However, future RCTs are necessary to validate the preventive effects of statin dosing in Parkinson’s disease.

Our study has several limitations. First, although a validation study of Japanese administrative medical data demonstrated the reliability of diagnoses in claim data,^44^ the use of ICD-10 codes to define the outcomes introduces the potential for misclassification. Although there were, to our knowledge, no studies validating the diagnostic accuracy of Parkinson’s disease in claim data by using electronic medical records in Japan, we conducted the sensitivity analysis using the ICD-10 and the medical records to define Parkinson’s disease, which was similar to previous studies.^14,17^ Although further study validating the diagnostic accuracy of Parkinson’s disease in administrative claim data would be needed in the future, we consider that the results of the sensitivity analysis consistently supported our main findings. Second, the mean follow-up duration in our study was approximately 2 years, which is relatively short compared with that in previous cohort studies.^14,18^ Long-term follow-up is crucial for the accurate assessment of the long-term effects of statins. Therefore, individuals with <180 or 365 days of follow-up were excluded from our sensitivity analyses, and the results remained unchanged. Third, given that this study was based on administrative claim data, we were unable to obtain serum cholesterol level or accurately assess medication adherence. Finally, our study was not adjusted for potential confounding factors. The absence of data on body mass index, smoking and alcohol consumption (potential confounders) may have led to an overestimation of the protective effects of statins in our study.^2^ We used the *E*-value to estimate the influence of the unmeasured confounders. If the unmeasured confounders were associated with both statin use and Parkinson’s disease risk with an OR of 2.66 or higher, the result was explained by unmeasured confounders.^29^ Future studies should focus on comprehensive adjustments for these confounding variables to provide a more accurate assessment of the effects of statins.

In our study, we observed a significant association between statin use and a reduced risk of Parkinson’s disease in older Japanese adults. Furthermore, individuals with low cumulative statin use had an increased risk of Parkinson’s disease compared with non-users. Meanwhile, high cumulative statin use showed a protective effect against the onset of Parkinson’s disease.

## Supplementary Material

fcae195_Supplementary_Data

## Data Availability

The authors do not have permission to share data. Inquiries about the data used in the present study can be addressed to the corresponding author. Researchers who are interested in LIFE study can refer to https://life.hcam.med.kyushu-u.ac.jp/.
