# Supplementary material for: Statin use and risk of Parkinson’s disease among older adults in Japan: a nested case–control study using the Longevity Improvement and Fair Evidence study
Source: Brain Commun. 2024 Jun 4;6(3):fcae195. doi: 10.1093/braincomms/fcae195 (PMC11184346; doi:10.1093/braincomms/fcae195)
Supplement: fcae195_Supplementary_Data [file fcae195_supplementary_data.docx]

| **Supplementary Table 1. Sample size and duration of receipt records for each municipality** | | | |
| --- | --- | --- | --- |
| **Municipality** | **Number of over all participants, n(%)** | **Number of analysis patients, n(%)** | **Data period for analysis (years)** |
| A | 670,191 (39.6) | 15,504 (27.6) | April 2017–May 2020 (3.2) |
| B | 327,414 (19.3) | 8,924 (15.9) | April 2015–March 2020 (5.0) |
| C | 154,132 (9.1) | 8,599 (15.3) | February 2015–December 2019 (4.9) |
| D | 114,582 (6.8) | 7,277 (13.0) | April 2014–December 2020 (6.8) |
| E | 69,643 (4.1) | 2,997 (5.3) | April 2014–September 2019 (5.5) |
| F | 49,034 (2.9) | 1,528 (2.7) | March 2015–December 2020 (5.8) |
| G | 42,209 (2.5) | 1,881 (3.3) | April 2015–July 2020 (5.3) |
| H | 38,924 (2.3) | 1,603 (2.9) | March 2015–June 2020 (5.3) |
| I | 33,183 (2.0) | 1,067 (1.9) | March 2015–March 2020 (5.1) |
| J | 31,741 (1.9) | 990 (1.8) | April 2014–March 2020 (6.0) |
| K | 30,227 (1.8) | 1,212 (2.2) | May 2015–March 2020 (4.9) |
| L | 29,936 (1.8) | 1,012 (1.8) | April 2014–October 2019 (5.6) |
| M | 23,502 (1.4) | 941 (1.7) | March 2015–May 2020 (5.3) |
| N | 22,860 (1.3) | 714 (1.3) | April 2015–March 2020 (5.0) |
| O | 21,960 (1.3) | 1,175 (2.1) | April 2014–March 2020 (6.0) |
| P | 19,743 (1.2) | 316 (0.6) | October 2015–September 2020 (5.0) |
| Q | 14,802 (0.9) | 446 (0.8) | April 2015–April 2020 (5.1) |
| Total | 1,694,083 (100) | 56,186 (100) |  |

| **Supplementary Table 2. Characteristics of Parkinson's disease cases and their controls for matched variables** | | | | |
| --- | --- | --- | --- | --- |
|  |  | **Participants, No. (%)** | | **Standardized difference** |
| **Characteristic** |  | **Controls** | **Case (Parkinson's disease)** |  |
| N |  | 46,789 | 9,397 |  |
| Sex, n (%) | |  |  | <0.01 |
|  | Men | 21,717 (46.4) | 4,362 (46.4) |  |
|  | Women | 25,072 (53.6) | 5,035 (53.6) |  |
| Age at cohort entry time, n (%) | |  |  | <0.01 |
|  | 65-74 | 13,242 (28.3) | 2,656 (28.3) |  |
|  | 75-84 | 23,143 (49.5) | 4,643 (49.4) |  |
|  | 85- | 10,404 (22.2) | 2,098 (22.3) |  |
| Municipality, n (%) | |  |  | 0.01 |
|  | A | 12,920 (27.6) | 2,584 (27.5) |  |
|  | B | 7,436 (15.9) | 1,488 (15.8) |  |
|  | C | 7,163 (15.3) | 1,436 (15.3) |  |
|  | D | 6,061 (13.0) | 1,216 (12.9) |  |
|  | E | 2,495 (5.3) | 502 (5.3) |  |
|  | F | 1,270 (2.7) | 258 (2.7) |  |
|  | G | 1,566 (3.3) | 315 (3.4) |  |
|  | H | 1,334 (2.9) | 269 (2.9) |  |
|  | I | 885 (1.9) | 182 (1.9) |  |
|  | J | 823 (1.8) | 167 (1.8) |  |
|  | K | 1,009 (2.2) | 203 (2.2) |  |
|  | L | 843 (1.8) | 169 (1.8) |  |
|  | M | 783 (1.7) | 158 (1.7) |  |
|  | N | 593 (1.3) | 121 (1.3) |  |
|  | O | 978 (2.1) | 197 (2.1) |  |
|  | P | 263 (0.6) | 53 (0.6) |  |
|  | Q | 367 (0.8) | 79 (0.8) |  |
| Cohort entry year, n (%) | |  |  | 0.01 |
|  | 2014 | 9,825 (21.0) | 1,965 (20.9) |  |
|  | 2015 | 21,117 (45.1) | 4,225 (45.0) |  |
|  | 2016 | 1,745 (3.7) | 360 (3.8) |  |
|  | 2017 | 12,969 (27.7) | 2,604 (27.7) |  |
|  | 2018 | 835 (1.8) | 178 (1.9) |  |
|  | 2019 | 276 (0.6) | 60 (0.6) |  |
|  | 2020 | 22 (0.1) | 5 (0.1) |  |

| **Supplementary Table 3. Definition and defined daily dose value of statin** | | | |
| --- | --- | --- | --- |
| **Key condition** | | **Definitions** | |
| **Exposure** | | **Drug name and Definitions** | **Defined daily dose (DDD, mg)** |
| Statin^a^ | Atorvastatin | Atorvastatin (C10AA05) Atorvastatin and Amlodipine (C10BX03)  Atorvastatin and Ezetimibe (C10BA05) | 20 |
|  | Fluvastatin | Fluvastatin (C10AA04) | 60 |
|  | Pitavastatin | Pitavastatin (C10AA08) | 2 |
|  | Pravastatin | Pravastatin (C10AA03) | 30 |
|  | Rosuvastatin | Rosuvastatin (C10AA07) Rosuvastatin and Ezetimibe (C10BA06) | 10 |
|  | Simvastatin | Simvastatin (C10AA01) | 30 |
| ^a^ Definition and defined daily dose value based on the WHO Collaborating Centre for Drug Statistics Methodology | | | |

| **Supplementary Table 4. The revised definition of PD in the sensitivity analysis** | | | |
| --- | --- | --- | --- |
| **Key condition** | **Definitions** | | |
| **Outcome** | **ICD-10** | **Parkinson's medication** | **ATC code^a^** |
| Parkinson's disease | G20 | Apomorphine | N04BC07 |
|  |  | Bromocriptine | N04BC01 |
|  |  | Cabergoline | N04BC06 |
|  |  | Dopa and dopa derivatives | N04BA |
|  |  | Dopamine agonists | N04BC |
|  |  | Droxidopa | C01CA27 |
|  |  | Entacapone | N04BX02 |
|  |  | Istradefylline | N04CX01 |
|  |  | Levodopa | N04BA01 |
|  |  | Levodopa and decarboxylase inhibitor | N04BA02 |
|  |  | Levodopa, decarboxylase inhibitor and COMT inhibitor | N04BA03 |
|  |  | Opicapone | N04BX04 |
|  |  | Pergolide | N04BC02 |
|  |  | Pramipexole | N04BC05 |
|  |  | Rasagiline | N04BD02 |
|  |  | Ropinirole | N04BC04 |
|  |  | Rotigotine | N04BC09 |
|  |  | Safinamide | N04BD03 |
|  |  | Selegiline | N04BD01 |
|  |  | Zonisamide | N03AX15 |
| ^a^ ATC index based on the WHO Collaborating Centre for Drug Statistics Methodology | | | |

| **Supplementary Table 5. Definition of comorbidities^a^** | |
| --- | --- |
| **Key condition** | **Definitions** |
| **Comorbidities** | **ICD-10** |
| Congestive heart failure (CHF) | I09.9, I11.0, I13.0, I13.2, I25.5, I42.0, I42.5, I42.6, I42.7, I42.8, I42.9, I43.0, I43.1, I43.2, I43.8, I50.0, I50.1, I50.9, P29.0 |
| Cardiac arrhythmias | I44.1, I44.2, I44.3, I45.6, I45.9, I47.0, I47.1, I47.2, I47.9, I48, I49.0, I49.1, I49.2, I49.3, I49.4, I49.5, I49.8, I49.9, R00.0, R00.1, R00.8, T82.1, Z45.0, Z95.0 |
| Valvular disease | A52.0, I05.0, I05.1, I05.2, I05.8, I05.9, I06.0, I06.1, I06.2, I06.8, I06.9, I07.0, I07.1, I07.2, I07.8, I07.9, I08.0, I08.1, I08.2, I08.3, I08.8, I08.9, I09.1, I09.8, I34.0, I34.1, I34.2, I34.8, I34.9, I35.0, I35.1, I35.2, I35.8, I35.9, I36.0, I36.1, I36.2, I36.8, I36.9, I37.0, I37.1, I37.2, I37.8, I37.9, I38, I39.0, I39.1, I39.2, I39.3, I39.4, I39.8, Q23.0, Q23.1, Q23.2, Q23.3, Z95.2, Z95.4 |
| Pulmonary circulation disorders | I26.0, I26.9, I27.0, I27.1, I27.2, I27.8, I27.9, I28.0, I28.8, I28.9 |
| Peripheral vascular disease | I70.0, I70.1, I70.2, I70.8, I70.9, I71.0, I71.1, I71.2, I71.3, I71.4, I71.5, I71.6, I71.8, I71.9, I73.1, I73.8, I73.9, I77.1, I79.0, I79.2, K55.1, K55.8, K55.9, Z95.8, Z95.9 |
| Hypertension | I10, I11.0, I11.9, I12.0, I12.9, I13.0, I13.1, I13.2, I13.9, I15.0, I15.1, I15.2, I15.8, I15.9 |
| Chronic pulmonary disease | I27.8, I27.9, J40, J41.0, J41.1, J41.8, J42, J43.0, J43.1, J43.2, J43.8, J43.9, J44.0, J44.1, J44.8, J44.9, J45.0, J45.1, J45.8, J45.9, J46, J47, J60, J61, J62.0, J62.8, J63.0, J63.1, J63.2, J63.3, J63.4, J63.5, J63.8, J64, J65, J66.0, J66.1, J66.2, J66.8, J67.0, J67.1, J67.2, J67.3, J67.4, J67.5, J67.6, J67.7, J67.8, J67.9, J68.4, J70.1, J70.3 |
| Diabetes | E10-E14 |
| Renal failure | I12.0, I13.1, N18.0, N18.8, N18.9, N19, N25.0, Z49.0, Z49.1, Z49.2, Z94.0, Z99.2 |
| Liver disease | B18.0, B18.1, B18.2, B18.8, B18.9, I85.0, I85.9, I86.4, I98.2, K70.0, K70.1, K70.2, K70.3, K70.4, K70.9, K71.1, K71.3, K71.4, K71.5, K71.7, K72.0, K72.1, K72.9, K73.0, K73.1, K73.2, K73.8, K73.9, K74.0, K74.1, K74.2, K74.3, K74.4, K74.5, K74.6, K76.0, K76.2, K76.3, K76.4, K76.5, K76.6, K76.7, K76.8, K76.9, Z94.4 |
| Cancer | C00-C97 |
| Psychosis | F20.0, F20.1, F20.2, F20.3, F20.4, F20.5, F20.6, F20.8, F20.9, F22.0, F22.8, F22.9, F23.0, F23.1, F23.2, F23.3, F23.8, F23.9, F24, F25.0, F25.1, F25.2, F25.8, F25.9, F28, F29, F30.2, F31.2, F31.5 |
| Depression | F20.4, F31.3, F31.4, F31.5, F32.0, F32.1, F32.2, F32.3, F32.8, F32.9, F33.0, F33.1, F33.2, F33.3, F33.4, F33.8, F33.9, F34.1, F41.2, F43.2 |
| ^a^ Defined by the codes of the International Classification of Diseases, 10th Edition | |

| **Supplementary Table 6. Association between specific types of statin use and risk of Parkinson's disease^a^** | | | |
| --- | --- | --- | --- |
|  |  | **No statin use^b^** | **Statin use^b^** |
| **Lipophilic statins** |  |  |  |
|  | Case/Control | 8,726/42,522 | 258/2,195 |
|  | Model 1 | Ref. | 0.57 (0.50-0.65) |
|  | Model 2 | Ref. | 0.62 (0.54-0.71) |
| **Hydrophilic statins** |  |  |  |
|  | Case/Control | 8,726/42,348 | 308/2,624 |
|  | Model 1 | Ref. | 0.57 (0.50-0.64) |
|  | Model 2 | Ref. | 0.62 (0.55-0.70) |
| Model 1: No adjustment. Model 2: Adjusted for CHF (yes or no), Cardiac arrhythmias (yes or no), Valvular disease (yes or no), Pulmonary circulation disorders (yes or no), Peripheral vascular disorders (yes or no), Hypertension (yes or no), Chronic pulmonary disease (yes or no), Diabetes (yes or no), Renal failure (yes or no), Liver disease (yes or no), Cancer (yes or no) , Psychoses (yes or no), Depression (yes or no), Number of months with outpatient visits and LTC needs.  ^a^ When exploring the relationship between lipophilic statins and the risk of PD, we excluded patients who had used other statins. Following this, we conducted an analysis on the cases and controls after applying a 1:5 re-matching procedure. This process was repeated for hydrophilic statins. ^b^ For lipophilic statins, the percentage of users was 4.6%, while the percentage of non-users was 95.4%. For hydrophilic statins, the percentage of users was 5.4%, while the percentage of non-users was 94.6%. | | | |

| **Supplementary Table 7. Association between statin use and risk of Parkinson's disease: Exclusion of individuals on multiple statins^a^** | | | | | |
| --- | --- | --- | --- | --- | --- |
|  |  |  | **No statin use^b^** | **Statin use^b^** | **P for interaction** |
| **All** |  | Case/Control | 8,726/41,353 | 566/4,896 |  |
|  |  | Model 1 | Ref. | 0.54 (0.50-0.60) |  |
|  |  | Model 2 | Ref. | 0.58 (0.53-0.64) |  |
| **Sex** |  |  |  |  | 0.59 |
|  | Men | Case/Control | 4,073/19,320 | 242/2,152 |  |
|  |  | Model 1 | Ref. | 0.53 (0.46-0.61) |  |
|  |  | Model 2 | Ref. | 0.56 (0.49-0.65) |  |
|  | Women | Case/Control | 4,653/22,033 | 324/2,744 |  |
|  |  | Model 1 | Ref. | 0.55 (0.49-0.62) |  |
|  |  | Model 2 | Ref. | 0.60 (0.53-0.68) |  |
| **Age** |  |  |  |  | 0.27 |
|  | 65-74 | Case/Control | 2,425/11,280 | 197/1,788 |  |
|  |  | Model 1 | Ref. | 0.51 (0.44-0.60) |  |
|  |  | Model 2 | Ref. | 0.56 (0.48-0.66) |  |
|  | 74-84 | Case/Control | 4,298/20,332 | 289/2,522 |  |
|  |  | Model 1 | Ref. | 0.54 (0.47-0.61) |  |
|  |  | Model 2 | Ref. | 0.57 (0.50-0.65) |  |
|  | 85- | Case/Control | 2,003/9,741 | 80/586 |  |
|  |  | Model 1 | Ref. | 0.66 (0.52-0.84) |  |
|  |  | Model 2 | Ref. | 0.70 (0.55-0.89) |  |
| Model 1: No adjustment. Model 2: Adjusted for CHF (yes or no), Cardiac arrhythmias (yes or no), Valvular disease (yes or no), Pulmonary circulation disorders (yes or no), Peripheral vascular disorders (yes or no), Hypertension (yes or no), Chronic pulmonary disease (yes or no), Diabetes (yes or no), Renal failure (yes or no), Liver disease (yes or no), Cancer (yes or no) , Psychoses (yes or no), Depression (yes or no), Number of months with outpatient visits and LTC needs.  ^a^ After excluding individuals who used multiple statins, we performed a 1:5 re-matching of cases and controls and then perform this analysis. ^b^ The percentage of statin users was 9.8%, while the percentage of non-users was 90.2%. | | | | | |

| **Supplementary Table 8. Association between total cumulative use of statin drugs and risk of Parkinson's disease: Exclusion of individuals on multiple statins^a^** | | | | | |
| --- | --- | --- | --- | --- | --- |
|  | **Category 1^b^** | **Category 2^b^** | **Category 3^b^** | **Category 4^b^** | **Category 5^b^** |
| TSDDs of statin | 0 | 1-30 | 31-90 | 91-180 | >180 |
| Case/Control | 8,726/41,353 | 224/1,011 | 127/899 | 99/925 | 116/2,061 |
| Model 1 | Ref. | 1.04 (0.90-1.21) | 0.66 (0.55-0.80) | 0.51 (0.41-0.62) | 0.26 (0.22-0.32) |
| Model 2 | Ref. | 1.15 (0.99-1.33) | 0.70 (0.58-0.85) | 0.55 (0.44-0.68) | 0.28 (0.23-0.34) |
| Model 1: No adjustment. Model 2: Adjusted for CHF (yes or no), Cardiac arrhythmias (yes or no), Valvular disease (yes or no), Pulmonary circulation disorders (yes or no), Peripheral vascular disorders (yes or no), Hypertension (yes or no), Chronic pulmonary disease (yes or no), Diabetes (yes or no), Renal failure (yes or no), Liver disease (yes or no), Cancer (yes or no) , Psychoses (yes or no), Depression (yes or no), Number of months with outpatient visits and LTC needs.  ^a^ After excluding individuals who used multiple statins, we performed a 1:5 re-matching of cases and controls and then perform this analysis. ^b^ The percentages of individuals in each group were as follows: 90.2% (Category 1); 2.2% (Category 2); 1.8% (Category 3); 1.8% (Category 4); and 4.0% (Category 5). | | | | | |

| **Supplementary Table 9. Association between statin use and risk of Parkinson's disease: Excluding short follow-up participants** | | |
| --- | --- | --- |
|  | **No statin use** | **Statin use** |
| **No exclusion^a^** | | |
| Case/Control | 8,726/41,282 | 671/5,507 |
| Model 1 | Ref. | 0.57 (0.53-0.62) |
| Model 2 | Ref. | 0.61 (0.56-0.66) |
| **Exclusion of less than 180 days Follow-up^b^** | | |
| Case/Control | 7,099/33,862 | 627/4,633 |
| Model 1 | Ref. | 0.64 (0.59-0.70) |
| Model 2 | Ref. | 0.67 (0.61-0.73) |
| **Exclusion of less than 365 days Follow-up^c^** | | |
| Case/Control | 5,737/27,539 | 581/3,948 |
| Model 1 | Ref. | 0.70 (0.64-0.77) |
| Model 2 | Ref. | 0.72 (0.65-0.79) |
| Model 1: No adjustment. Model 2: Adjusted for CHF (yes or no), Cardiac arrhythmias (yes or no), Valvular disease (yes or no), Pulmonary circulation disorders (yes or no), Peripheral vascular disorders (yes or no), Hypertension (yes or no), Chronic pulmonary disease (yes or no), Diabetes (yes or no), Renal failure (yes or no), Liver disease (yes or no), Cancer (yes or no) , Psychoses (yes or no), Depression (yes or no), Number of months with outpatient visits and LTC needs.  ^a^ The percentage of statin users was 11.0%, while the percentage of non-users was 89.0%. ^b^ The percentage of statin users was 11.4%, while the percentage of non-users was 88.6%. ^c^ The percentage of statin users was 12.0%, while the percentage of non-users was 88.0%. | | |

| **Supplementary Table 10. Association between total cumulative use of statin drugs and risk of Parkinson's disease: Excluding short follow-up participants** | | | | | |
| --- | --- | --- | --- | --- | --- |
|  | **Category 1** | **Category 2** | **Category 3** | **Category 4** | **Category 5** |
| TSDDs of statin | 0 | 1-30 | 31-90 | 91-180 | >180 |
| **No exclusion^a^** | | | | | |
| Case/Control | 8,726/41,282 | 237/915 | 144/923 | 134/1,113 | 156/2,556 |
| Model 1 | Ref. | 1.22 (1.05-1.41) | 0.73 (0.61-0.87) | 0.57 (0.47-0.68) | 0.29 (0.24-0.34) |
| Model 2 | Ref. | 1.30 (1.12-1.52) | 0.77 (0.64-0.92) | 0.62 (0.52-0.75) | 0.30 (0.25-0.35) |
| **Exclusion of less than 180 days Follow-up^b^** | | | | | |
| Case/Control | 7,099/33,862 | 208/751 | 131/777 | 132/924 | 156/2,181 |
| Model 1 | Ref. | 1.32 (1.13-1.54) | 0.80 (0.66-0.96) | 0.68 (0.56-0.81) | 0.34 (0.29-0.40) |
| Model 2 | Ref. | 1.38 (1.18-1.63) | 0.82 (0.68-1.00) | 0.74 (0.61-0.89) | 0.34 (0.29-0.41) |
| **Exclusion of less than 365 days Follow-up^c^** | | | | | |
| Case/Control | 5,737/27,539 | 179/630 | 121/672 | 126/779 | 155/1,867 |
| Model 1 | Ref. | 1.36 (1.15-1.61) | 0.86 (0.71-1.05) | 0.77 (0.64-0.93) | 0.39 (0.33-0.47) |
| Model 2 | Ref. | 1.41 (1.19-1.68) | 0.86 (0.71-1.06) | 0.82 (0.67-1.00) | 0.40 (0.33-0.47) |
| Model 1: No adjustment. Model 2: Adjusted for CHF (yes or no), Cardiac arrhythmias (yes or no), Valvular disease (yes or no), Pulmonary circulation disorders (yes or no), Peripheral vascular disorders (yes or no), Hypertension (yes or no), Chronic pulmonary disease (yes or no), Diabetes (yes or no), Renal failure (yes or no), Liver disease (yes or no), Cancer (yes or no) , Psychoses (yes or no), Depression (yes or no), Number of months with outpatient visits and LTC needs.  ^a^ The percentages of individuals in each group were as follows: 89.0% (Category 1); 2.0% (Category 2); 2.0% (Category 3); 2.2% (Category 4); and 4.8% (Category 5). ^b^ The percentages of individuals in each group were as follows: 88.6% (Category 1); 2.1% (Category 2); 2.0% (Category 3); 2.3% (Category 4); and 5.0% (Category 5). ^c^ The percentages of individuals in each group were as follows: 88.0% (Category 1); 2.1% (Category 2); 2.1% (Category 3); 2.4% (Category 4); and 5.4% (Category 5). | | | | | |

| **Supplementary Table 11. Characteristics of Parkinson's disease cases and their controls in the sensitivity analysis employing the revised definition of PD** | | | | |
| --- | --- | --- | --- | --- |
|  |  | **Participants, No. (%)** | | **Standardized difference** |
| **Characteristic** | | **Controls** | **Case (Parkinson's disease)** |  |
| N |  | 29,607 | 5,942 |  |
| Sex, n (%) | |  |  | <0.01 |
|  | Male | 14,170 (47.9) | 2,845 (47.9) |  |
|  | Female | 15,437 (52.1) | 3,097 (52.1) |  |
| Age at cohort entry time, n (%) | |  |  | <0.01 |
|  | 65-74 | 8,526 (28.8) | 1,708 (28.7) |  |
|  | 75-84 | 15,348 (51.8) | 3,078 (51.8) |  |
|  | 85- | 5,733 (19.4) | 1,156 (19.5) |  |
| Municipality, n (%) | |  |  | 0.01 |
|  | A | 8,600 (29.0) | 1,720 (28.9) |  |
|  | B | 4,721 (15.9) | 945 (15.9) |  |
|  | C | 4,146 (14.0) | 830 (14.0) |  |
|  | D | 3,680 (12.4) | 738 (12.4) |  |
|  | E | 1,538 (5.2) | 309 (5.2) |  |
|  | F | 861 (2.9) | 174 (2.9) |  |
|  | G | 999 (3.4) | 201 (3.4) |  |
|  | H | 810 (2.7) | 162 (2.7) |  |
|  | I | 672 (2.3) | 139 (2.3) |  |
|  | J | 505 (1.7) | 101 (1.7) |  |
|  | K | 746 (2.5) | 150 (2.5) |  |
|  | L | 425 (1.4) | 85 (1.4) |  |
|  | M | 478 (1.6) | 97 (1.6) |  |
|  | N | 473 (1.6) | 97 (1.6) |  |
|  | O | 534 (1.8) | 107 (1.8) |  |
|  | P | 197 (0.7) | 40 (0.7) |  |
|  | Q | 222 (0.7) | 47 (0.8) |  |
| Cohort entry year, n (%) | |  |  | 0.01 |
|  | 2014 | 5,935 (20.0) | 1,187 (20.0) |  |
|  | 2015 | 13,199 (44.6) | 2,640 (44.4) |  |
|  | 2016 | 1,108 (3.7) | 229 (3.9) |  |
|  | 2017 | 8,612 (29.1) | 1,729 (29.1) |  |
|  | 2018 | 569 (1.9) | 116 (2.0) |  |
|  | 2019 | 172 (0.6) | 38 (0.6) |  |
|  | 2020 | 12 (0.1) | 3 (0.1) |  |
| Comorbidities at look-back period (yes), n (%) | |  |  |  |
|  | Congestive heart failure | 4,564 (15.4) | 1,105 (18.6) | 0.08 |
|  | Cardiac arrhythmias | 4,766 (16.1) | 1,077 (18.1) | 0.05 |
|  | Valvular disease | 1,868 (6.3) | 377 (6.3) | <0.01 |
|  | Pulmonary circulation disorders | 158 (0.5) | 23 (0.4) | 0.02 |
|  | Peripheral vascular disorders | 3,279 (11.1) | 791 (13.3) | 0.07 |
|  | Hypertension | 16,815 (56.8) | 3,411 (57.4) | 0.01 |
|  | Chronic pulmonary disease | 5,970 (20.2) | 1,181 (19.9) | 0.01 |
|  | Diabetes | 1,958 (6.6) | 438 (7.4) | 0.03 |
|  | Renal failure | 1,341 (4.5) | 347 (5.8) | 0.06 |
|  | Liver disease | 5,834 (19.7) | 1,167 (19.6) | <0.01 |
|  | Cancer | 4,067 (13.7) | 818 (13.8) | <0.01 |
|  | Psychosis | 942 (3.2) | 490 (8.2) | 0.22 |
|  | Depression | 1,969 (6.7) | 869 (14.6) | 0.26 |
| LTC needs, n (%) | |  |  | 0.30 |
|  | No | 24,561 (83.0) | 4,193 (70.6) |  |
|  | Support 1–2 | 1,506 (5.1) | 476 (8.0) |  |
|  | Care 1 | 1,048 (3.5) | 380 (6.4) |  |
|  | Care 2 | 832 (2.8) | 353 (5.9) |  |
|  | Care 3 | 649 (2.2) | 235 (4.0) |  |
|  | Care 4 | 611 (2.1) | 193 (3.2) |  |
|  | Care 5 | 400 (1.4) | 112 (1.9) |  |
| Number of months with outpatient visit, n (%) | |  |  | 0.13 |
|  | 0 | 430 (1.5) | 129 (2.2) |  |
|  | 1 | 132 (0.4) | 52 (0.9) |  |
|  | 2 | 210 (0.7) | 56 (0.9) |  |
|  | 3 | 374 (1.3) | 120 (2.0) |  |
|  | 4 | 1,007 (3.4) | 239 (4.0) |  |
|  | 5 | 1,720 (5.8) | 447 (7.5) |  |
|  | 6 | 25,734 (86.9) | 4,899 (82.4) |  |

| **Supplementary Table 12. Association between statin use and risk of Parkinson's disease in the sensitivity analysis employing the revised definition of PD** | | | | | |
| --- | --- | --- | --- | --- | --- |
|  |  |  | **No statin use^a^** | **Statin use^a^** | **P for interaction** |
| **All** |  | Case/Control | 5,522/26,068 | 420/3,539 |  |
|  |  | Model 1 | Ref. | 0.56 (0.50-0.62) |  |
|  |  | Model 2 | Ref. | 0.58 (0.52-0.64) |  |
| **Sex** |  |  |  |  | 0.81 |
|  | Men | Case/Control | 2,661/12,643 | 184/1,527 |  |
|  |  | Model 1 | Ref. | 0.57 (0.49-0.67) |  |
|  |  | Model 2 | Ref. | 0.59 (0.50-0.69) |  |
|  | Women | Case/Control | 2,861/13,425 | 236/2,012 |  |
|  |  | Model 1 | Ref. | 0.54 (0.47-0.63) |  |
|  |  | Model 2 | Ref. | 0.57 (0.49-0.66) |  |
| **Age** |  |  |  |  | 0.72 |
|  | 65-74 | Case/Control | 1,557/7,249 | 151/1,277 |  |
|  |  | Model 1 | Ref. | 0.55 (0.46-0.65) |  |
|  |  | Model 2 | Ref. | 0.53 (0.44-0.64) |  |
|  | 74-84 | Case/Control | 2,856/13,460 | 222/1,888 |  |
|  |  | Model 1 | Ref. | 0.55 (0.48-0.64) |  |
|  |  | Model 2 | Ref. | 0.58 (0.50-0.67) |  |
|  | 85- | Case/Control | 1,109/5,359 | 47/374 |  |
|  |  | Model 1 | Ref. | 0.61 (0.44-0.83) |  |
|  |  | Model 2 | Ref. | 0.63 (0.46-0.86) |  |
| Model 1: No adjustment. Model 2: Adjusted for CHF (yes or no), Cardiac arrhythmias (yes or no), Valvular disease (yes or no), Pulmonary circulation disorders (yes or no), Peripheral vascular disorders (yes or no), Hypertension (yes or no), Chronic pulmonary disease (yes or no), Diabetes (yes or no), Renal failure (yes or no), Liver disease (yes or no), Cancer (yes or no) , Psychoses (yes or no), Depression (yes or no), Number of months with outpatient visits and LTC needs.  ^a^ The percentage of statin users was 11.1%, while the percentage of non-users was 88.9%. | | | | | |

| **Supplementary Table 13. Association between total cumulative use of statin drugs and risk of Parkinson's disease in the sensitivity analysis employing the revised definition of PD** | | | | | |
| --- | --- | --- | --- | --- | --- |
|  | **Category 1^a^** | **Category 2^a^** | **Category 3^a^** | **Category 4^a^** | **Category 5^a^** |
| TSDDs of statin | 0 | 1-30 | 31-90 | 91-180 | >180 |
| Case/Control | 5,522/26,068 | 145/612 | 85/638 | 91/701 | 99/1,588 |
| Model 1 | Ref. | 1.10 (0.92-1.33) | 0.63 (0.50-0.79) | 0.61 (0.49-0.76) | 0.29 (0.24-0.36) |
| Model 2 | Ref. | 1.15 (0.95-1.39) | 0.66 (0.52-0.83) | 0.62 (0.50-0.78) | 0.30 (0.24-0.37) |
| Model 1: No adjustment. Model 2: Adjusted for CHF (yes or no), Cardiac arrhythmias (yes or no), Valvular disease (yes or no), Pulmonary circulation disorders (yes or no), Peripheral vascular disorders (yes or no), Hypertension (yes or no), Chronic pulmonary disease (yes or no), Diabetes (yes or no), Renal failure (yes or no), Liver disease (yes or no), Cancer (yes or no) , Psychoses (yes or no), Depression (yes or no), Number of months with outpatient visits and LTC needs.  a The percentages of individuals in each group were as follows: 88.9% (Category 1); 2.0% (Category 2); 2.2% (Category 3); 2.2% (Category 4); and 4.7% (Category 5). | | | | | |

| **Supplementary Table 14. Association between statin use and risk of Parkinson's disease stratified by cohort entry year** | | | | | |
| --- | --- | --- | --- | --- | --- |
|  |  |  | **No statin use** | **Statin use** | **P for interaction** |
| **Cohort entry year** | |  |  |  | 0.52 |
|  | 2014-2016 | Case/Control | 6033/28437 | 517/4250 |  |
|  |  | Model 1 | Ref. | 0.57 (0.52-0.63) |  |
|  |  | Model 2 | Ref. | 0.58 (0.49-0.69) |  |
|  | 2017-2020 | Case/Control | 2693/12845 | 154/1257 |  |
|  |  | Model 1 | Ref. | 0.60 (0.54-0.66) |  |
|  |  | Model 2 | Ref. | 0.64 (0.54-0.77) |  |
| Model 1: No adjustment. Model 2: Adjusted for CHF (yes or no), Cardiac arrhythmias (yes or no), Valvular disease (yes or no), Pulmonary circulation disorders (yes or no), Peripheral vascular disorders (yes or no), Hypertension (yes or no), Chronic pulmonary disease (yes or no), Diabetes (yes or no), Renal failure (yes or no), Liver disease (yes or no), Cancer (yes or no) , Psychoses (yes or no), Depression (yes or no), Number of months with outpatient visits and LTC needs. | | | | | |

| **Supplementary Table 15. Association between total cumulative use of statin drugs and risk of Parkinson's disease stratified by cohort entry year** | | | | | | | | |
| --- | --- | --- | --- | --- | --- | --- | --- | --- |
|  |  |  | **Category 1** | **Category 2** | **Category 3** | **Category 4** | **Category 5** | **P for interaction** |
| TSDDs of statin | | | 0 | 1-30 | 31-90 | 91-180 | >180 |  |
| **Cohort entry year** | |  |  |  |  |  |  | 0.80 |
|  | 2014-2016 | Case/Control | 6033/28437 | 181/673 | 101/664 | 100/778 | 135/2135 |  |
|  |  | Model 1 | Ref. | 1.26 (1.07-1.49) | 0.71 (0.58-0.88) | 0.60 (0.49-0.74) | 0.30 (0.25-0.35) |  |
|  |  | Model 2 | Ref. | 1.10 (0.82-1.47) | 0.78 (0.56-1.08) | 0.48 (0.34-0.69) | 0.24 (0.15-0.37) |  |
|  | 2017-2020 | Case/Control | 2693/12845 | 56/242 | 43/259 | 34/335 | 21/421 |  |
|  |  | Model 1 | Ref. | 1.32 (1.11-1.57) | 0.74 (0.60-0.92) | 0.65 (0.53-0.81) | 0.30 (0.25-0.36) |  |
|  |  | Model 2 | Ref. | 1.26 (0.93-1.71) | 0.82 (0.59-1.16) | 0.55 (0.38-0.79) | 0.25 (0.16-0.40) |  |
| Model 1: No adjustment. Model 2: Adjusted for CHF (yes or no), Cardiac arrhythmias (yes or no), Valvular disease (yes or no), Pulmonary circulation disorders (yes or no), Peripheral vascular disorders (yes or no), Hypertension (yes or no), Chronic pulmonary disease (yes or no), Diabetes (yes or no), Renal failure (yes or no), Liver disease (yes or no), Cancer (yes or no) , Psychoses (yes or no), Depression (yes or no), Number of months with outpatient visits and LTC needs. | | | | | | | | |

**Supplementary Figure 1. Graphical description of the study design**


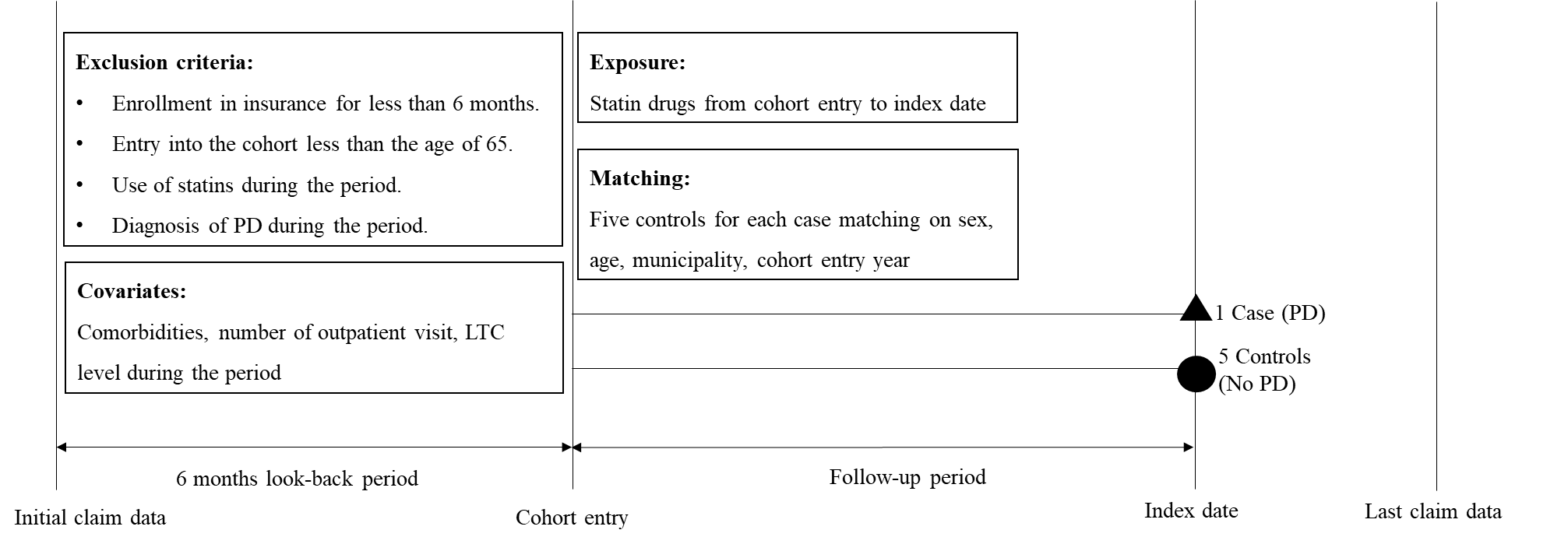


This was a population-based, nested, case-control study. For each case, five event-free controls were randomly selected based on their identification dates (index dates). The matching criteria were age at the cohort entry date, sex, municipality (17 municipalities), and year of cohort entry (2014–2020).

PD: Parkinson’s disease (PD); LTC: long-term care

Initial/last claim record referred to the first/last medical record covering any medical service or treatment.
